# Complete Genome Sequence of *Prevotella intermedia* Strain 17-2

**DOI:** 10.1128/genomeA.00951-15

**Published:** 2015-08-20

**Authors:** Takayuki Nambu, Kazuyoshi Yamane, Hugo Maruyama, Chiho Mashimo, Takeshi Yamanaka

**Affiliations:** Department of Bacteriology, Osaka Dental University, Hirakata, Osaka, Japan

## Abstract

*Prevotella intermedia*, a Gram-negative black-pigmented anaerobic rod, is frequently isolated from not only periodontal pockets but also purulent infections. We report here the complete genome sequence of *P. intermedia* strain 17-2, which is a non-exopolysaccharide-producing variant obtained from exopolysaccharide (EPS)-producing *P. intermedia* strain 17 stock culture.

## GENOME ANNOUNCEMENT

Exopolysaccharides (EPSs) play a variety of important roles in biofilm formation, with which numerous bacteria express their virulence to the host (1). Evidence in the literature suggests that bacteria with biofilm-forming ability have enormous advantages, particularly in establishing persistent infections (2). Therefore, the management of biofilm infection is a worldwide health care issue (3, 4). We have previously reported that *Prevotella intermedia* strain 17 isolated from a chronic periodontitis lesion produces a large amount of mannose-rich EPS and forms biofilm. The strain 17 induced highly noticeable abscess lesions in mice at 10^7^ CFU, but non-EPS-producing *P. intermedia* strains ATCC 25611 or 17-2, a naturally occurring variant of the strain 17, required 100-fold more bacteria (10^9^ CFU) in order to induce detectable abscess lesions (5). The complete genome sequence of strain 17 has been determined by the Institute for Genomic Research (TIGR) and released through the NCBI under GenBank accession numbers CP003502 and CP003503.

Here, we sequenced the whole genome of *P. intermedia* strain 17-2 that cannot produce either EPS or biofilm-like structures. A single colony of the strain 17-2 was inoculated into trypticase soy broth supplemented with 0.5% yeast extract, hemin (5 mg/liter), l-cystine (400 mg/liter), and vitamin K_1_ (10 mg/liter), and grown in an anaerobic chamber. Genomic DNA was extracted using a MagExtractor kit (TOYOBO). A 20-kb SMRTbell library was prepared, and the genome was sequenced using a PacBio RS II system (Pacific Biosciences) on a single-molecule real-time (SMRT) cell using PacBio P6-C4 chemistry.

*De novo* assembly of 127,972 reads with a mean length of 6,598 bp using the Hierarchical Genome Assembly Process (HGAP) algorithm in the SMRT Analysis version 2.3 (6) resulted in two closed circular chromosomes of 606,227-bp and 2,131,046-bp in size with average coverages of 194.0× and 214.6×, respectively. The genome has a G+C content of 43.5% and was then annotated by RAST (Rapid Annotation using Subsystem Technology) version 2.0 (7), which successfully identified 2,510 coding sequences, as well as 61 RNA sequences. Of these, 32% of the annotated coding sequences fell within 262 subsystems available in the RAST database. The complete genome sequence of strain 17-2 will contribute to the better understanding of the mechanisms of EPS productivity and biofilm-mediated infection of this organism.

### Nucleotide sequence accession numbers.

The genome sequence of *Prevotella intermedia* strain 17-2 has been deposited in the DDBJ/EMBL/GenBank database under the accession numbers AP014925 and AP014926.
